# Tracheotomy Performed by M. Ricord

**Published:** 1849-11

**Authors:** 


*Tracheotomy performed by M. Ricord.*—It is with much pleasure that we extract, from *L' Union Medicale*, the following details, which do great honor to M. Ricord's generous decision of conduct.

A man, thirty-three years of age, was admitted, on the 5th of June, 1849, into the Hôpital du Midu, with tertiary syphilitic symptoms. His left shoulder and arm were affected with a tubercular eruption, and the scrotum presented the same condylomatous aspect. There was, besides, great dyspnœa and loss of voice. These symptoms were referred to an indurated urethral chancre, contracted in 1838, for which no mercurial treatment had been used. M. Ricord diagnosed syphilitic tubercles in the larynx, similar to those appearing on the shoulder and scrotum, resting his diagnosis on the following reasons:—the tubercles had existed these four years, and the hoarseness and dyspnœa three and a half, though the latter had become aggravated only two months ago. No member of the patient's family, either living or dead, had ever had any symptoms of consumption, and the examination of the chest (very imperfect, from a noisy breathing,) presented only a slight dulness under the left clavicle. The man was put on the usual treatment of tertiary symptoms—viz: on iodide of potassium; and it was hoped that the well-known and rapid effects of this salt on tertiary syphilis would obviate the necessity of tracheotomy. But the very next day after admission, the patient was seized, towards the evening, with such fearful fits of dyspnœa, that he walked about the gardens and yards the whole night, gasping for breath. At seven o'clock in the morning, when M. Ricord began his visit, it was evident that no resource but tracheotomy was left; and though the eminent surgeon was ill of cholerine, and his pupils were entreating him to allow one of his colleagues to act in his stead, he made preparations to operate at once. The patient could hardly draw his breath when he was brought in, and scarcely had M. Ricord began his incision, when the subject was perceived to be a corpse. This was an awful moment, and the bystanders thought all was over with the poor man, when M. Ricord rapidly cut away four rings of the trachea from the cricoid cartilage downwards; and setting aside every feeling of repugnance, he applied his mouth to the aperture, rendered very repulsive by a recent blister, drew with his breath the blood and pus which were obtructing the trachea, and replaced these obstructing fluids by a vigorous insuffluation of air into the patient's lungs. This being repeated rapidly fifteen or twenty times, restored life to a corpse which revived amidst the deafening applause of a numerous concourse of pupils. Thoughtless of his besmeared face, and mouth impregnated with purulent matter, the skilful and generous surgeon would not think of himself until he was quite sure that his patient was beyond the danger of suffocation.

Those who witnessed this scene will hardly ever forget the prompt and truly philanthropic conduct of M. Ricord. The tracheal aperture was kept open by curved forceps, fixed by bands passing under the axilla; and the patient was put upon emollient fumigations and iodide of potassium. On the 9th he could take broth, had no fever, and spoke. On the 11th he smoked a pipe by stealth, spoke, and breathed somewhat through the nose; M. Ricord replaced the forceps by a canula. On the 14th every one was astonished to witness the extraordinary effects of the iodide; the patient laid the canula aside, put on his cravat, and breathed easily through the nose. On the 22d he spoke and breathed with ease. The eventual cure was now no longer doubtful, and the patient was ordered to go on with the same treatment.—*London Lancet.*

